# Future Me, a Prospection-Based Chatbot to Promote Mental Well-Being in Youth: Two Exploratory User Experience Studies

**DOI:** 10.2196/74411

**Published:** 2025-07-18

**Authors:** Martin Dechant, Eva Lash, Sarah Shokr, Ciarán O'Driscoll

**Affiliations:** 1 University College London, 1-19 Torrington Place, London, WC1E 7HB, United Kingdom, +44 (0) 20 7679 1897; 2 University of Oxford, Oxford, United Kingdom

**Keywords:** chatbot, artificial intelligence, prospection, mental health, student well-being, stress, anxiety

## Abstract

**Background:**

Digital interventions have been proposed as a solution to meet the growing demand for mental health support. Large language models (LLMs) have emerged as a promising technology for creating more personalized and adaptive mental health chatbots. While LLMs generate responses based on statistical patterns in training data rather than through conscious reasoning, they can be designed to support important psychological processes. Prospection—the ability to envision and plan for future outcomes—represents a transdiagnostic process altered across various mental health conditions that could be effectively targeted through such interventions. We designed “Future Me,” an LLM-powered chatbot designed to facilitate future-oriented thinking and promote goal pursuit using evidence-based interventions including visualization, implementation intentions, and values clarification.

**Objective:**

This study aims to understand how users engage with Future Me, evaluate its effectiveness in supporting future-oriented thinking, and assess its acceptability across different populations, with particular attention to postgraduate students’ stress management needs. We also seek to identify design improvements that could enhance the chatbot’s ability to support users’ mental well-being.

**Methods:**

In total, 2 complementary studies were conducted. Study 1 (n=20) examined how postgraduate students used Future Me during a single guided session, followed by semistructured interviews. Study 2 (n=14) investigated how postgraduate students interacted with Future Me over a 1-week period, with interviews before and after usage. Both studies analyzed conversation transcripts and interview data using thematic analysis to understand usage patterns, perceived benefits, and limitations.

**Results:**

Across both studies, participants primarily engaged with Future Me to discuss career or education goals, personal obstacles, and relationship concerns. Users valued Future Me’s ability to provide clarity around goal-setting (85% of participants), its nonjudgmental nature, and its 24/7 accessibility (58%). Future Me effectively facilitated self-reflection (80%) and offered new perspectives (70%), particularly for broader future-oriented concerns. However, both studies revealed limitations in the chatbot’s ability to provide personalized emotional support during high-stress situations, with participants noting that responses sometimes felt formulaic (50%) or lacked emotional depth. Postgraduate students specifically emphasized the need for greater context awareness during periods of academic stress (58%). Overall, 57% of requests occurred outside office hours, dropping from 40 on day 1 to 12 by day 7.

**Conclusions:**

Future Me demonstrates promise as an accessible tool for promoting prospection skills and supporting mental well-being through future-oriented thinking. However, effectiveness appears context-dependent, with prospection techniques more suitable for broader life decisions than acute stress situations. Future development should focus on creating more adaptive systems that can adjust their approach based on the user’s emotional state and immediate needs. Rather than attempting to replicate human therapy entirely, chatbots like Future Me may be most effective when designed as complementary tools within broader support ecosystems, offering immediate guidance while facilitating connections to human support when needed.

## Introduction

The rising demand for mental health support has led to a significant gap between those seeking help and available resources. Approximately 11% of people in the United States seek psychotherapy annually [1], with demand consistently outstripping supply. Additionally, postgraduate students face unique mental health challenges, including academic workload, financial insecurity, and social isolation, which can negatively impact their well-being [2,3]. Digital interventions have emerged as accessible and effective early intervention or prevention techniques to address mental health problems, potentially reducing the burden on traditional services and providing support when human alternatives are unavailable.

Digital mental health interventions face significant challenges, including low adherence rates [4], financial and logistical barriers to accessibility [5], and a lack of personalization. Many users disengage from digital tools due to generic, repetitive responses that fail to adapt to individual emotional states and stress levels [6,7]. Addressing these challenges is crucial for developing effective, scalable mental health tools that align with users’ needs, particularly for younger populations like students who may be more receptive to digital solutions.

Recent advancements in large language models (LLMs) have transformed the landscape of digital mental health interventions. Unlike earlier rule-based chatbots, LLM-based conversational agents can gather situation-specific information through natural conversation to provide personalized and context-specific interventions. These chatbots could improve equity and accessibility [8] by personalizing interventions, reminding users, tracking progress, and offering multilingual and adaptable communication options for users of diverse backgrounds and technology literacy.

Therapeutic chatbots have been used across a broad spectrum of mental health conditions, focusing on specific issues such as anxiety, depression, and autism [1]. Most of these interventions position the chatbot as a different entity, like a digital therapist. However, LLM-powered chatbots may have the capability to enact more convincing alternative roles, offering novel therapeutic approaches beyond traditional digital therapy paradigms.

One promising application of LLM chatbots in mental health involves enabling goal pursuit, an aspect of functioning compromised in various mental health conditions, including depression, addiction, and schizophrenia [9]. The extent to which we can imagine, plan for, and enjoy thinking about the future affects the decisions we make now. This cognitive process (prospection) involves the ability to envision and plan for possible future outcomes, allowing individuals to adaptively integrate information from the past and present to simulate potential futures [10]. When used appropriately, prospection enables goal pursuit, facilitates flexible decision-making, and contributes to maintaining a coherent sense of personal identity [11].

Research suggests that individuals with stronger identification with their future self tend to make better decisions in daily life, such as exercising more frequently [12], staying on the right side of the law [13], achieving better academic grades [14], experiencing greater well-being [15], and attaining superior financial outcomes [16]. These benefits are understood as a consequence of the reduced difference between the future and present self, which results in improved inter-temporal decision-making—with individuals more likely to select “should” behaviors (eg, exercising, saving money, going to bed early) over “want” behaviors (eg, staying on the sofa, spending money, staying up late) [17]. Within the context of mental health, future-oriented therapies aim to promote goal-directed behavior [18]. Therapeutic approaches have long recognized the significance of targeting prospection to improve well-being. Cognitive behavioral therapy uses techniques that aim to readjust clients’ overly pessimistic predictions by addressing cognitive distortions like catastrophizing and fortune-telling [19] and incorporates Socratic questioning to help clients develop more accurate predictions about their future [20]. Other prospective therapeutic practices with promising results include future-directed therapy [21], mental contrasting and implementation intentions [22], and goal-setting and planning techniques [23].

Using an LLM chatbot may further facilitate these future-oriented approaches by enabling users to engage with their future self, developing the capacity for intertemporal decision-making. These conversational agents may offer users support and guidance as and when they need it. However, due to the inherent opacity of LLMs, it is crucial to evaluate how they provide such support and, more significantly, how individuals interact with them.

While chatbots have been shown to provide valuable support through their 24/7 availability and nonjudgmental nature [24], most research on mental health interventions has relied on retrospective self-reports, which may overlook moment-to-moment variations in stress levels and emotional states [25]. Few studies have explored on-demand, chatbot-based interventions that incorporate prospection-based techniques, particularly for student populations who may benefit significantly from improved future-oriented thinking. Prior research points to several well-documented risk factors faced by postgraduate students, including academic pressures [26], financial concerns [27,28], future uncertainty [29], and social isolation [30], where prospective thinking may help to overcome these factors and improve the overall mental well-being.

To address these gaps, we developed “Future Me,” a chatbot prototype that uses GPT-3.5 to facilitate future-oriented thinking by simulating a conversation with the user’s future self. The interaction with Future Me aims to stimulate a future-oriented mindset by strengthening people’s ability and motivation to consider their future and fostering identification with their future self.

This paper presents 2 complementary studies examining the usability, acceptability, and potential efficacy of Future Me:

- Study 1 investigates how postgraduate participants use Future Me during a single guided session, followed by interviews to assess their experience.
- Study 2 examines how postgraduate students interact with Future Me over a 1-week period, with interviews before and after usage to understand expectations and experiences.

Together, these studies aim to answer the following research questions: (1) How do people use Future Me, and what types of support do they seek from it? (2) In what ways does Future Me enable or support the process of future-oriented thinking? (3) How are the acceptability and usability of Future Me perceived by different user groups? (4) What are the specific perceptions and needs of postgraduate students regarding chatbot-based support for stress management? (5) How can chatbot design be improved to better address users’ mental well-being needs?

By exploring these questions, we aim to provide comprehensive insights into the potential of LLM-based chatbots for promoting prospection skills and supporting mental well-being and identify key design considerations for developing more effective digital mental health interventions.

## Methods

### Design Overview

Both studies used qualitative approaches to evaluate the usability and effectiveness of Future Me, a chatbot designed to facilitate future-oriented thinking. Study 1 focused on a single guided session, while Study 2 examined sustained usage over 1 week. Both studies used semistructured interviews and analyzed conversation transcripts between users and Future Me. We used the SRQR (Standards for Reporting Qualitative Research) checklist [31] for reporting our study (see Checklist 1).

### Participants

Study 1 comprised a convenience sample of 20 postgraduate students (12 female, 6 male, and 2 nonbinary), aged 21‐40 (mean 25.1, SD 4.39) years. Study 2 consisted of 14 postgraduate students (3 men and 11 women) aged 22 to 33 years (mean 23.71, SD 2.73) years.

Eligibility criteria included being 18 years or older, the ability to read and understand English, being based in the United Kingdom, having a personal mobile phone, and not currently experiencing mental health problems.

Participants were recruited for both studies through social media postings and advertisements on campus, outside of any clinical context.

### The Future Me Prototype

Both studies used the same core Future Me chatbot prototype, which used GPT-3.5 to facilitate future-oriented thinking based on principles of Mental Contrasting with Implementation Intentions. Mental contrasting involves imagining a future desired outcome and reflecting on present obstacles that impede its attainment. By making the connection between current reality and the desired future more explicit, individuals gain a deeper understanding of the challenges they may face and the feasibility of their goals.

Implementation intentions involve “if... then...” thinking, which promotes consideration of anticipated obstacles, planning to overcome these obstacles, and, consequently, more successful goal pursuit. Together, these therapeutic techniques have been shown to consistently aid goal attainment and promote a wide range of beneficial outcomes.

Future Me was also designed to use Socratic questioning, a key feature of cognitive behavioral therapy, involving a series of graded questions to guide thought toward a therapeutic goal strategically. The aim of bringing these therapeutic techniques together was to enable users to link their current behavior and future consequences, facilitating skills in decision-making, goal-setting, and behavior change.

In both studies, participants interacted with Future Me by sending SMS messages to a registered phone number. Each message was stored on a local server, processed to create prompts for the GPT-3.5 model, and responses were sent back to the user’s phone. The system used an adaptive prompt creation mechanism, which refined the initial manual prompt as conversations progressed using each user’s message history to ’train’ subsequent responses. This approach merges previous responses from the system and questions from the user into an expanded prompt sent back to the GPT-3.5 model to ensure that the responses can include previously discussed topics.

Participants were instructed that the prototype is not intended for crisis support, and external support resources (eg, contact information for crisis support services) were handed out during the onboarding for the experiments in case participants needed support. Due to the low-level nature of the prototype, Future Me was not able to sense or detect signs of distress (eg, measuring the user’s heart rate).

### Procedures

#### Study 1

The intervention involved a 15-minute interaction with Future Me via SMS text messaging on their phone in a quiet location chosen by the participants. The duration was considered long enough to have a meaningful interaction and assess the user experience in line with similar research [32]. Before involvement, participants were instructed that Future Me was designed to be open and self-directed, with the primary aim of facilitating thinking about the future and assisting in the exploration of prospective decisions, goals, and problems. To have relevant material for the conversation, participants were asked to come up with approximately 3 obstacles or decisions they were currently facing. Following the interaction, semistructured interviews were conducted with participants via video conference to assess dimensions of usability, acceptability, and signs of preliminary efficacy.

#### Study 2

The study was conducted in 3 phases.

##### Preinteraction Interviews

Semistructured interviews lasting approximately 45 minutes were conducted web-based via videoconference. These explored students’ stress experiences, coping strategies, and expectations from digital mental health tools. Participants discussed their stress management routines, daily stressors, responses to the Perceived Stress Scale (PSS)-10, and attitudes toward digital mental health tools and chatbots.

##### Interaction With Future Me

After the first interviews, participants received instructions for using the Future Me chatbot. They were informed that the chatbot was not intended for crisis intervention and were provided with external support resources. Participants were asked to interact with Future Me over 1 week. After 7 days, they completed a second survey that included the PSS-10 and questions about their experiences with Future Me. Participants were required to use the system at least once in the beginning but were otherwise free to use it as often as they wished.

##### Follow-Up Interviews

The final phase involved 25‐ to 30-minute follow-up interviews to assess the usability and effectiveness of the Future Me chatbot and explore changes in perceptions of digital mental health tools.

### Measurements

#### Common Measures

Both studies used semistructured interviews to capture individuals’ reactions to and experiences with Future Me. Interview questions were designed to assess dimensions of usability, acceptability, and preliminary efficacy. Additionally, both studies recorded and analyzed the conversation transcripts between participants and Future Me.

#### Study 2: Additional Measures

##### PSS-10

Perceived stress was measured using the PSS-10 [33], a widely used tool with high reliability. This 10-item scale assesses how often individuals have recently felt their lives were overwhelming, uncontrollable, and overloaded using a 5-point Likert scale. For this study, the timeframe was modified to measure stress over the past week rather than the past month, to correspond with the period participants interacted with Future Me.

##### Interaction Measurements

For each SMS message participants sent to the chatbot, the timestamp and message content were stored, along with the chatbot’s response, allowing for analysis of usage patterns throughout the week.

### Data Analysis

#### Conversation Analysis

In Study 1, conversations between participants and Future Me were analyzed using framework analysis with NVivo [34]. The thematic framework used to code participant inputs was based on the COM-B model for behavior change [35], which identifies 3 key requirements needed for behavior change: capabilities (C), opportunities (O), and motivations (M). The transtheoretical model of behavior change was used to develop the framework for coding Future Me’s responses, which posits that behavior change occurs through stages: pre-contemplation, contemplation, preparation, action, maintenance, and termination [36].

In Study 2, a reflexive bottom-up thematic analysis was adopted to analyze conversation transcripts. The data were codified into key themes, which were then reviewed and consolidated into overarching themes. To ensure reliability, a second researcher independently reviewed the transcripts using a top-down analysis based on the initial codebook created by the first researcher.

#### Interview Analysis

In Study 1, interview transcripts were thematically analyzed using the method outlined by Braun and Clarke [37]. Coding was inductive, based on the frequency of sentiments across interviews.

In Study 2, a similar thematic analysis approach was used to assist in data coding and theme development. The interaction logs with Future Me were also analyzed to examine the average number and timing of interactions, assessing whether patterns changed throughout the week.

#### Reflexivity Statement

The researchers’ backgrounds in psychology, human-computer interaction, and computer science could influence both chatbot design and analytical approach, potentially favoring technological solutions for mental health. Personal experiences with academic stress and mental health apps could also shape our assumptions about user needs. We implemented reflexive practices to mitigate these potential biases.

### Ethical Considerations

Both studies were approved by the research ethics committee of University College London (ethics approval IDs: CEHP/2024/597 (Study 1) and UCLIC_2023_003_Dechant_Manning (Study 2) and followed a similar procedure for providing informed consent. All participants provided informed consent electronically before the beginning of each study. The consent form detailed the study purpose, procedures, potential risks and benefits, data privacy protection, and voluntary participation. Participants in both studies received a £20 (US $26.86) gift card after completion of the whole study.

All participant data were deidentified before analysis. Identifiable information, including phone numbers and email addresses, was removed before the analysis. Data were stored on encrypted servers compliant with institutional and GDPR (General Data Protection Regulation) guidelines.

## Results

### Study 1

#### User Inputs

Our analysis revealed that goals fell into 3 core categories: “Career/education,” “Personal,” and “Relationships.” The “Career/education” theme occupied more than half of user inputs, aligning well with the COM-B model, with most inputs falling into the capability component. This was divided into 2 separate codes to recognize that participants inquired about both their professional capability (eg, skills, education, and work experience) and their emotional capacity to reach these professional capacities.

The second largest theme was “Personal,” in which users expressed current obstacles in their lives and asked for advice to overcome these issues. Most inputs focused on internal factors, specifically psychological and behavioral aspects, falling under the “Capability” category, followed by “Motivation” and “Combination,” with only a few falling into “Opportunity.”

Within “Relationships,” most inputs fell into the “Motivation” category, in which individuals described relationship dynamics and nuance that informed their desire to commit to a behavior change or decision. The remaining inputs were coded under “Feasibility,” which consisted of a combination of their capability and opportunity to action a behavior related to a personal relationship.

The final theme, “Anthropomorphizing,” fell outside the COM-B framework and consisted of comments indicating the degree to which users were humanizing Future Me. Most frequently, participants used conversational markers and social cues similar to a typical human-human conversation (eg, introducing themselves, saying thank you). The other codes in “Anthropomorphizing” suggested that users were either keenly aware of Future Me being an AI and therefore did not use typical conversational cues (“expressionless tone”) or directly questioned Future Me’s capacity and limits (“curiosity”).

Multimedia Appendix 1 summarizes the themes participants discussed during the usage of Future Me.

#### Future Me Outputs

The transtheoretical model was used to categorize Future Me’s responses according to the presumed stage of change of the user. Responses were suggestive of users being in 2 out of the 6 stages—“Contemplation” and “Preparation.”

Under “Contemplation,” users identified a problem or goal and were evaluating whether and how to pursue it. Future Me primarily responded by “Inquiring” about the context of their decision, “Suggesting” information or techniques they could use, and “Evaluating” by highlighting relevant factors and nuances affecting a decision.

The second stage seen was “Preparation,” in which users had committed to a decision and were given action-based steps. There were significantly fewer outputs in this stage compared to “Contemplation” (42 vs 176).

'Recognition’ was present in both stages and formed its own theme. In this theme, Future Me reflected information received back to the user through “Acknowledgment” (neutral recognition or statement of understanding) and “Positive reinforcement” (recognition with a hopeful and encouraging tone).

Multimedia Appendix 1 outlines the themes of the answers generated by Future Me collected during the conversations with the chatbot.

#### Participant Interviews

Thematic analysis of the interviews revealed several key themes.

#### Initial Perceptions of Future Me

Participants had 3 main expectations before interacting with Future Me: “Novelty of AI as a therapeutic tool,” “Interest” in testing AI capabilities or exploring personal issues, and “Skepticism” that Future Me would provide generic or vague responses.

#### Topics of Conversation

Participants engaged in multidimensional conversations, combining practical and professional elements with personal preferences and motivations. Many discussed only professional goals, believing Future Me was best suited for objective and unemotional advice and expressed skepticism about using it for personal matters due to perceived limitations of an AI not being human.

#### Positive Perceptions of Future Me

The main positive aspect mentioned was that Future Me provided “Clarity and practicality” (85%), helping with idea mapping, exploring options, and evaluating pros and cons. Participants also valued how Future Me facilitated personal reflection by asking questions rather than providing answers (80%), finding it “unbiased” and “nonjudgmental.”

Additionally, 70% reported that Future Me provided new information and perspectives, particularly about career paths and techniques for personal issues. Participants found Future Me “reaffirming and consolidating” (65%) prior knowledge regarding personal obstacles, making them feel “reassured” and more “confident.”

In “Comparison to human conversation” (65%), participants described the interaction as “easy” and “natural,” noting that Future Me exhibited features traditionally associated with being human, such as “emotional intelligence” and “empathy.” The “Text message format” (50%) was appreciated for its accessibility and comfort, making the conversation feel less formal and more human.

#### Negative Perceptions of Future Me

The main limitation mentioned was “User-dependence” (70%), with users feeling they had to take responsibility for guiding the conversation. The main criticism (60%) was that Future Me provided “formulaic” or overly general responses, though some acknowledged that advice from human sources can also be vague.

Half the participants thought Future Me was “lacking something human” (50%), finding answers less meaningful due to a lack of “compassion,” “intention,” or “empathy.” Some participants (45%) felt uneasy about the “Conversation flow,” particularly the fluctuating response time that disrupted normal conversation patterns, leading to feelings described as “disconcerting” and “frustrated.”

Multimedia Appendix 1 outlines the positive and negative themes found in the user feedback collected after the interaction with Future Me.

### Study 2: Results

#### General Perceptions and Needs for Stress Management From Chatbots

Multimedia Appendix 1 outlines the themes from the preintervention interview transcripts.

#### Trust and Emotional Openness

While students found chatbots useful for immediate advice and practical support, they highlighted a lack of emotional depth as a significant limitation, particularly in high-stress situations. Participants expressed that, during moments of heightened stress, genuine compassion and nuanced emotional support were critical qualities they felt chatbots could not provide, leading to a reluctance among some to rely on chatbots for deep emotional support.

Concerns over data privacy also influenced students’ trust in chatbots, particularly regarding integration within university systems. Several participants expressed hesitation about the possibility of their conversations being accessed by university staff, fearing potential academic or professional implications.

#### Role and Effectiveness

The potential for chatbots to detect early signs of stress was viewed positively, with students noting that early warnings could help prevent stress from escalating. However, there was a widespread perception that chatbots lacked the emotional depth necessary for high-stress situations, leading to a reluctance among some students to use chatbots for emotional support.

#### Personalization and Contextual Awareness

Students expressed a need for chatbots to provide personalized support tailored to their specific academic stressors, envisioning chatbots having detailed background knowledge of their courses for more targeted advice. They highlighted the need for support appropriately tailored to their current stress levels, emphasizing that different situations require different responses.

#### Support Preferences

Students consistently valued the 24/7 accessibility of chatbots, highlighting the convenience of having support available at any time. Anonymity was also a significant factor, with many noting that it encouraged more openness in sharing experiences. The non-judgmental nature of chatbot interactions was cited as reducing the pressure to filter thoughts, making it easier to discuss sensitive topics.

#### Expressions of Stress and Support Sought From a Chatbot in Real Time

Multimedia Appendix 1 outlines the themes of participants’ inputs during conversations with Future Me.

#### Academic Stress and Time Management Advice

Postgraduate students, particularly those in their final term, experienced significant stress related to academic demands, with a strong focus on dissertation work. They sought practical, immediate relief through quick advice and activities, appreciating Future Me’s swift SMS responses for short-term stress management.

#### Support to Tackle Negative Emotions and Low Motivation

Students reported emotional distress linked to academic workload and personal challenges. They found self-reflective questions useful for future uncertainties but frustrating when applied to immediate academic stress, as they felt these questions were time-consuming and added to their emotional burden.

#### Future Uncertainty and Advice on How to Handle It

International students expressed heightened stress about future uncertainties, particularly concerning finances, student loans, and job prospects before visa expiration. This theme was more pronounced among international students compared to their domestic peers.

#### Advice About Social Relationships

Although not as prominently discussed, social relationships were indirectly mentioned in the context of overall stress. The focus was primarily on academic and future-related concerns rather than detailed issues with social relationships.

#### Future Me Engagement Metrics

**Figure 1.**
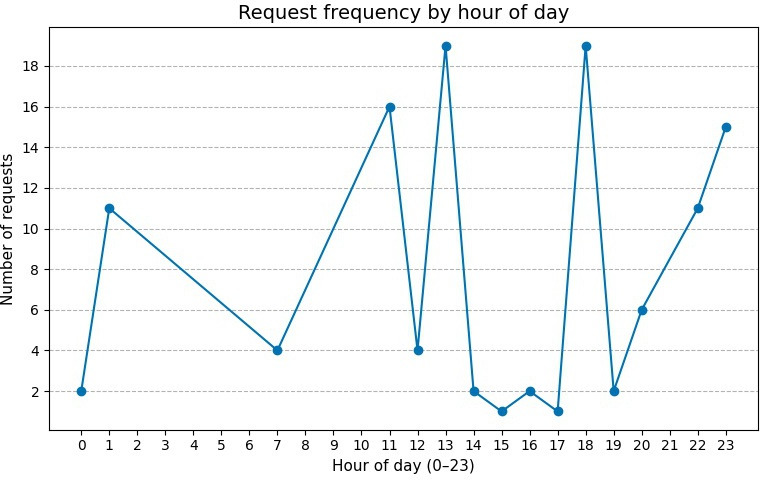
Frequency distribution of requests per hour over the full experiment.

**Figure 2.**
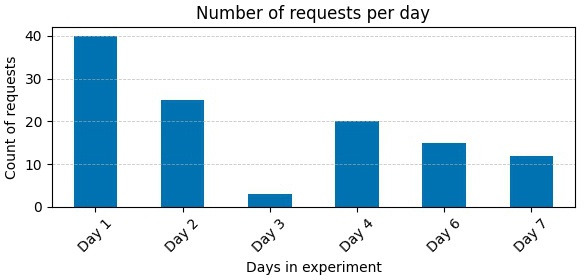
Distribution of requests sent to the chatbot via SMS per day.

Participants’ interactions with Future Me were spread throughout the day, with 49 out of 115 chatbot interactions occurring between 9 AM and 5 PM, and the remaining 66 occurring outside the typical work hours. Figure 1 shows the distribution of requests over time. Participants sent most of the requests at the beginning of the experiment (N=40), with a linear decrease in the interaction over the week (see Figure 2).

A paired-samples *t* test of PSS scores before (mean 20.25, SD 5.96) and after (mean 17.67, SD 6.51) using Future Me for 1 week showed no significant difference (*t*_11_=1.765, *P*=.105).

#### User Engagement, Specific Perceptions, and Experiences of Using Chatbots for Stress Management

Multimedia Appendix 1 outlines the themes revealed from the follow-up interview transcripts.

#### Accessibility and Convenience

Postgraduate students found Future Me highly accessible and convenient, particularly outside working hours. They appreciated its personalized interactions and conversational tone, comparing the experience to speaking with a friend. In total, 3 students noted that simply expressing their current situations to Future Me sometimes helped relieve stress, similar to venting to friends.

#### Personalized and Relatable

Students valued the practical advice Future Me offered but felt there was a gap in its ability to provide empathetic support during high-stress times. They appreciated Future Me’s reflective questions for broader issues like future uncertainty but preferred these questions to focus more on understanding emotions during immediate concerns. When reflective questions were used during acute stress, students found the responses scripted and impersonal, leading to frustration.

#### Need for Better Balance Between Advice and Emotional Support

Several students found it difficult to express themselves clearly to Future Me during high-stress periods, noting that humans can often fill in blanks using body language and facial expressions. Students emphasized that they would be more open to using chatbots during high-stress periods if there were other ways to interpret their emotions, suggesting voice systems to make expression easier.

#### Increased Demand for Context Awareness

Students called for Future Me to be more context-aware and responsive to their immediate emotional states. They felt that integrating Future Me with professional mental health services could provide a more comprehensive support system, allowing the chatbot to assist with less serious issues while referring students to appropriate professionals for specialized support.

#### Connection With Human Support Systems

Students viewed Future Me as a valuable complement to human support rather than a replacement. They saw a need for integrating the chatbot with professional mental health services and believed it could help navigate the complexity of university services by referring them to appropriate professionals. While generally willing to share their chatbot data to streamline this process, they preferred those referrals be handled sensitively to avoid feeling dismissed.

## Discussion

### Summary of Findings

Both studies provide valuable insights into how users interact with Future Me, a chatbot designed to facilitate future-oriented thinking through simulated conversations with one’s future self. While Study 1 focused on a single guided session with non-clinical adults, Study 2 examined sustained usage over 1 week among postgraduate students. These preliminary investigations point toward several promising directions for further development and research.

First, both studies demonstrate that users primarily engaged with Future Me to discuss career and education goals, personal obstacles, and, to a lesser extent, social relationship concerns. The prevalence of career-related topics suggests that users find the chatbot particularly suitable for objective, goal-oriented discussions that benefit from structured future thinking. This aligns with findings from both studies and prior work [38] that participants viewed Future Me as less suitable for emotionally complex or high-stress situations that might require more nuanced human support.

Second, both studies revealed that Future Me effectively facilitated 2 key processes: guided introspective questioning and collaborative solution generation. Analysis of conversation transcripts in Study 1 showed that Future Me’s responses primarily fell under the “contemplation” stage of the transtheoretical model, helping users gather information, evaluate options, and move toward preparation for action. Similarly, Study 2 found that Future Me encouraged reflection and offered new perspectives, particularly valuable for addressing broader concerns like future uncertainty.

Third, users across both studies appreciated similar qualities in Future Me. Nonclinical participants in Study 1 and students in Study 2 both valued the chatbot’s ability to provide clarity around goal setting [39], its nonjudgmental nature, and its accessibility through SMS text messaging [40,41]. Both groups also found value in Future Me’s ability to facilitate personal reflection without the influence of others’ perspectives or judgment [42,43].

However, both studies also identified similar limitations. Participants in both samples noted that Future Me sometimes provided formulaic or generic responses that lacked personalization. Users in both studies felt that the chatbot lacked something inherently human, despite its ability to generate conversational responses. This was particularly evident during high-stress moments in Study 2, where students found reflective questions, although important for managing stress, frustrating when seeking immediate emotional support.

### Implications for Design

#### Balancing Reflective and Directive Approaches

A key direction emerging from both studies is the need for chatbots to balance reflective questioning with more directive guidance based on the user’s emotional state and immediate needs. While the Socratic questioning and prospective thinking techniques used by chatbots, like Future Me, were generally well-received in prior work [44], our studies indicate that these approaches may be less effective during periods of acute stress or when users seek immediate practical advice.

Our findings strongly advocate for mental health chatbot designers to adopt a more adaptive and nuanced approach that considers both the user’s emotional state and their surrounding context, including cultural factors [45]. This perspective aligns with prior research demonstrating that the chatbot’s communication style significantly influences user acceptance [46]. Whenever a system detects signs of high stress or anxiety using various techniques [47], the chatbot could temporarily shift from reflection-promoting questions to more direct, solution-focused responses that address immediate concerns. Once the acute stress has been acknowledged and addressed, the chatbot could then gradually reintroduce reflective elements to promote longer-term thinking and behavior change.

#### Enhancing Personalization and Context Awareness

Both studies highlighted the importance of personalization in maintaining user engagement. Study 1 found that 70% of participants appreciated when Future Me provided new information specific to their situation, while Study 2 revealed that students wanted the chatbot to understand their academic context and tailor support accordingly.

Future iterations of mental health chatbots could substantially enhance personalization by implementing a layered approach to user understanding. By developing progressive profiling capabilities, these systems could build increasingly nuanced user models over time, remembering important details from previous conversations and creating a more continuous experience. This historical awareness would allow the chatbot to reference past concerns or achievements, creating a sense of ongoing relationships rather than disconnected interactions. Alongside this individual history, context integration would enable chatbots to recognize the broader circumstances affecting users’ well-being. For instance, a system aware of university examination periods or job application deadlines could proactively adjust its support approach during these high-stress times, offering relevant coping strategies without users needing to explain their situation repeatedly.

### Integration With Human Support Systems

Study 2 particularly emphasized the potential value of integrating chatbots within broader support systems. Participating students viewed Future Me as a complement to human support rather than a replacement, suggesting that mental health chatbots could serve as an initial point of contact that guides users toward appropriate professional services when needed.

This finding has important implications for institutional implementation of mental health chatbots, particularly within university settings. Rather than positioning chatbots as stand-alone solutions, designers should consider how these tools can form part of a comprehensive support ecosystem. This might include features that facilitate warm handoffs to human professionals, provide information about available resources, or offer continuous support between professional sessions.

### Theoretical Implications

#### Prospection and Digital Mental Health Support

Both studies contribute to our understanding of how digital tools can facilitate prospection skills. The findings suggest that LLM-based chatbots can effectively support aspects of future-oriented thinking by helping users clarify goals, identify obstacles, and develop implementation intentions. This aligns with existing literature on the benefits of prospection for mental well-being and extends it by demonstrating the potential of AI-mediated conversations to strengthen future self-identification.

However, the studies also reveal nuances in how prospection techniques should be applied in digital interventions. While these approaches appear beneficial for addressing broader life decisions and goals, they may be less effective during moments of acute stress when immediate relief is the priority. This suggests that prospection-based digital interventions should be designed with sensitivity to timing and emotional context.

#### Anthropomorphism and Therapeutic Alliance

Both studies provide insights into how users anthropomorphize and form relationships with AI chatbots. Study 1 found that 65% of participants compared their interaction with Future Me to human conversation, noting qualities like “emotional intelligence” and “empathy.” Similarly, Study 2 revealed that students compared talking to Future Me to speaking with a friend.

These findings suggest that users can form a type of therapeutic alliance with chatbots, even while remaining aware of their nonhuman nature. The nonjudgmental quality of chatbot interactions appears particularly valuable, potentially reducing barriers to self-disclosure that might exist in human relationships. However, both studies also found that users perceived limitations in the emotional depth and authenticity of the chatbot, highlighting the complex nature of human-AI therapeutic relationships.

### Ethical Concerns

The use of artificial intelligence in mental health is challenging traditional practices and offering novel approaches to address existing barriers. However, these innovations also raise significant ethical concerns that clinicians and researchers must carefully consider. One major issue is bias in AI models, which are often trained on datasets that underrepresent diverse populations. This can lead to neglect of users’ cultural and personal needs, potentially compromising the quality and relevance of support provided. For example, in the context of Future Me, overlooking cultural nuances may harm users’ sense of identity and well-being [48]. Privacy is another critical concern. AI-based mental health tools inherently handle sensitive personal data, raising risks around data security and user confidentiality [49]. Moreover, safeguarding is essential in AI systems used for mental health. Studies have shown that systems like ChatGPT can, under certain prompt conditions, generate harmful content [50,51]. Despite ongoing efforts to enhance safety mechanisms, AI may still fail to detect subtle cues related to self-harm or distress, posing risks to vulnerable users.

### Limitations and Future Directions

Several limitations should be considered when interpreting the findings from these studies. First, both studies used relatively small, non-representative samples, limiting the generalizability of findings. Study 1 used convenience sampling with non-clinical adults in the United Kingdom, while Study 2 focused specifically on postgraduate students, with international students overrepresented.

Second, the controlled research context may have influenced how participants engaged with Future Me. In both studies, participants were aware they were part of a research trial, which may have affected their privacy concerns and usage patterns. Future research should examine more naturalistic usage in nonresearch contexts to better understand how users would engage with the chatbot in everyday life.

Third, the timeframes in both studies were relatively short—a single session in Study 1 and 1 week in Study 2. While Study 2 provided valuable insights into sustained engagement, longer-term studies are needed to understand how usage patterns evolve over time and whether benefits are maintained. Previous research has indicated that online mental health interventions often suffer from low retention rates, making longitudinal studies particularly important for evaluating practical utility.

Fourth, Future Me was only tested with English as the input language. Future work may explore other languages and multimodal ways, such as voice and visual inputs, to increase the accessibility of the approach.

Fifth, *Future Me* relied on GPT-3.5, a model known to exhibit various biases—gender, racial, cultural, linguistic, and ideological [52]. Users could not choose a more suitable or nuanced model tailored to their needs, leaving them exposed to these underlying biases of GPT 3.5. Additionally, *Future Me* could only gather information from user input during conversations, lacking any mechanism to collect essential contextual data such as gender, age, or personal preferences. This limitation further increased the risk of biased or inappropriate responses.

Future research should address these limitations while pursuing several promising avenues. Longer-term studies tracking users over 8‐12 weeks would yield more comprehensive insights into engagement patterns, attrition rates, and the sustainability of benefits beyond initial interactions. Additionally, expanding testing to diverse populations, including those with clinical conditions and from varied demographic backgrounds, would enhance our understanding of how Future Me’s benefits and usage patterns might differ across different contexts and user needs. The limitations of text-only communication, particularly during high-stress moments, could be addressed through multimodal interfaces, making it not only easier to communicate with the device but also giving more data to detect signs of mental needs such as high stress [53] or anxiety [54]. Such approaches might better capture emotional nuances and reduce the burden on users when articulating complex feelings. Integration studies represent another crucial direction, examining how Future Me could function within existing mental health ecosystems to complement professional services and therapies, potentially serving as a bridge between self-help and clinical intervention. Finally, comparative effectiveness research directly measuring Future Me against other digital interventions and traditional therapeutic approaches would provide valuable insights into its unique contributions and limitations. Such comparisons would help situate Future Me within the broader landscape of mental health support tools and clarify its most appropriate applications and user groups. Together, these research directions would substantially advance our understanding of how LLM-based chatbots can effectively support mental well-being through prospection-focused interventions.

### Conclusions

The 2 studies presented in this paper provide complementary insights into the potential of LLM-powered chatbots for promoting prospection skills and supporting mental well-being. Future Me demonstrates promise as an accessible, non-judgmental tool that can facilitate reflection, provide practical guidance, and help users connect their present actions to future outcomes. The findings suggest that such chatbots can fulfill a valuable supportive role, particularly when human alternatives are unavailable or when users prefer anonymous, pressure-free interaction.

However, the studies also highlight important limitations and design considerations. Future Me’s effectiveness appears context-dependent, with prospection techniques more suitable for broader life decisions than acute stress situations. Users appreciated the chatbot’s accessibility and reflective capabilities but noted limitations in emotional depth and personalization that affected sustained engagement.

These findings suggest that future development of mental health chatbots should focus on creating more adaptive, context-aware systems that can adjust their approach based on the user’s emotional state and immediate needs. Rather than attempting to replicate human therapy entirely, chatbots like Future Me may be most effective when designed as complementary tools within broader support ecosystems, offering immediate, accessible reflection and guidance while facilitating connections to human support when needed.

As LLM technology continues to advance, the potential for chatbots to provide increasingly personalized and adaptive mental health support will likely grow. Future Me represents an important early exploration of how these technologies can be harnessed to promote prospection skills and support well-being, providing a foundation for continued research and development in this promising field.

## Supplementary material

10.2196/74411 Multimedia Appendix 1 Summary of all quotes in tables.

10.2196/74411 Checklist 1 SRQR (Standards for Reporting Qualitative Research) checklist.

## Abbreviations

GDPR: General Data Protection Regulation
LLM: large language model
PSS-10: Perceived Stress Scale
SRQR: Standards for Reporting Qualitative Research
