# Supplementary material for: Future Me, a Prospection-Based Chatbot to Promote Mental Well-Being in Youth: Two Exploratory User Experience Studies
Source: JMIR Form Res. 2025 Jul 18;9:e74411. doi: 10.2196/74411 (PMC12296204; doi:10.2196/74411)
Supplement: Multimedia Appendix 1 [file formative-v9-e74411-s001.docx]

# Appendix

Table 1 Themes of the input phrases of users collected during the conversations with FutureMe

| **Theme**  **(bold) /code** | **Description** | **N of inputs** | **Example (codes only)** |
| --- | --- | --- | --- |
| **Career or education** | **Users ask for advice about their career or education** | **103** |  |
| Emotional capability | Users express and/or question their emotional capacity to  approach and accomplish work/education goals | 23 | *“How do I get over my fear of rejection when I apply to jobs.”* |
| Professional capability | Users ask about how to increase their professional capability  to achieve their goal or share their relevant background | 22 | *“How do you go about promoting your skills to a potential  employee even if you are unsure about your ability.”* |
| Opportunity | Users raise questions about specific actions they  can take to reach their professional goal | 30 | *“What is the best city in Europe to pursue a bioscience PhD.”* |
| Motivation | Users share motivations underlying decisions being  made about career/education | 28 | *“I want to do something productive where it feels my intellect and time is valued. I want to make enough money to live comfortably.”* |
|  |  |  |  |
| **Personal** | **Users share personal problems or goals** | **48** |  |
| Capability | Factors affecting users’ capability to pursue a goal, such as  internal psychological and behavioural factors. | 17 | *“But I am worried about money.  I want to have 4 kids but how will I be able to afford it.”* |
| Opportunity | External factors affecting the users goals | 3 | *“What if I can't use a breathing technique, for example, because I am at work.”* |
| Motivation | Internal factors that drive or inhibit action, such as internal conflicts | 14 | *“Ok, I am also struggling to decide between finding contentedness within the present or demanding  more and pushing myself to achieve greater things.”* |
| Combination | Users ask broad question about how to approach a personal goal, usually an opening question | 14 | *“How do I stop eating so many crisps.”* |
|  |  |  |  |
| **Relationship** | **Users present a relationship concern** | **13** |  |
| Feasibility | Users state concerns or ask about the feasibility of them taking action, combination of capability and opportunity | 3 | *“I don't know how to support my sister at the moment, she's going to have an operation soon.”* |
| Motivation | Users present their motivations in the form of  internal dialogue about a relationship concern | 10 | *“There's too much exchange of personal information.  this only exists within the paid hr. I find it strange.”* |
|  |  |  |  |
| **Anthropomorphising** | **The degree to which users humanise Future Me** | **35** |  |
| Conversational tone | Users interact with Future Me using a variety of typically  human-human social conversation markers  (e.g. introduce themselves, say please/thank you,  acknowledge Future Me’s help/advice) | 24 | *“Ok, thank you. I will have a conversation."* |
| Curiosity | Users ask Future Me about its ability or personal opinion | 8 | *“What are the things that you can do”* |
| Expressionless tone | Users not acknowledging Future Me, not using punctuation  or put little effort into normal social cues that facilitate  human-human conversation | 3 | *“Yes ask me questions."* |

Table 2:Themes of the answers generated by Future Me collected during the conversations with the chat bot.

| **Theme (bold) /code** | **Description** | **N of inputs** | **Example (codes only)** |
| --- | --- | --- | --- |
| **Contemplation** | **Assists the user in the contemplation stage. Users are aware of the need to change and are evaluating it** | **176** |  |
| Enquiring | Enquires about the context of the user’s obstacle or goal, including questions about previous steps taken and personal motivation | 91 | “What steps have you taken towards becoming a published writer so far?” |
| Evaluating | Guides the user through weighing up relevant factors for a decision | 47 | “The answer to this question is highly dependent on your location and lifestyle preferences” |
| Suggesting | Outlines various potential options, behaviours and/or  decisions a user might want to take to pursue a goal or tackle a problem | 48 | If you're interested in meeting new people and potentially finding a romantic partner, travelling solo can be a great way to do so. Additionally, participating in group activities.” |
| Preparation | Assists the user in the preparation stage. By this stage,  users have committed to a decision and Future Me  assists them by giving them lists of action steps | 42 | “Sure, here are some steps you can take to break the smoking habit: 1. Identify your reasons for quitting.” |
|  |  |  |  |
| **Recognition** | **Expresses a recognition of something stated by the user** | **58** |  |
| Acknowledgement | Acknowledges the user’s emotional state, personal preferences and motivations | 37 | “It's natural to feel frustrated when expectations aren't met.” |
| Positive reinforcement | Highlights a positive from what the user has shared with them or encourages the user | 21 | “It's great that you're taking initiative to improve the condition of your gym.” |

Table 3: Themes of the discussions between Future Me and the participants

| **Theme** | **Description** | **Frequency of presence** | **Example (codes only)** |
| --- | --- | --- | --- |
| **Initial reaction to Future Me** |  |  |  |
| Novelty of AI as a  therapeutic tool | Mentions that Future Me is the first therapeutic  chatbot that they have interacted with or that  they have only interacted with chatbots  in non-therapeutic contexts | 100% | “Customer service chat like with your bank and things like that but no, I haven't had too much experience with them” |
| Interest | Mentions feeling interested, excited,  curious or intrigued about interacting  with Future Me | 65% | “I'm just kind of interested in what they are capable of and how useful they could be” |
| Scepticism | Mentions feeling sceptical about  Future Me’s capabilities | 45% | “I thought it would be somewhat of a trivial exercise if I'm being honest,  because I know how these work, the large language models yield quite generic answers which are not,  which just cannot be tailored because of the data set and so I just thought it would yield generic advice” |
|  |  |  |  |
| **Topics of conversation** |  |  |  |
| Balancing personal and professional | Mentions that their conversation with Future Me  combined elements of their professional and  personal life | 100% | “How can I overcome my personal insecurities, like personal things that I don't like about myself in order to make better decisions in  the future and like psychological obstacles that I'm facing towards my career” |
| Professional life | Reports that their conversation with Future Me  was about their work, such as career advice  or helping to overcome work procrastination. | 100% | “It gave me, like other suggestions for getting into quantitative finance,  basic but really useful suggestions like highlight age analysis, get certificates, emphasise experience” |
| Personal life | Reports that their conversation with Future Me  was about an obstacle in their personal life  such as their health or relationships. | 80% | “It gave me good ideas about the weed, like identifying your reasons for quitting, like ok, maybe I should do that and like set a quit date.” |

Table 4:Positive themes found in the user feedback collected after the interaction with Future Me

| **Theme** | **Description** | **Frequency of presence** | **Example (codes only)** |
| --- | --- | --- | --- |
| **Positive perceptions of Future Me** |  |  |  |
| Clarity and practicality | Mentions Future Me helping them find clarity  of thought or clarity regarding actionable steps | 85% | *“So even though I knew that there's a tension, maybe, between career goals and financial aspirations, to have it laid out concretely in text was probably quite useful,  because it means it's not just  a hypothesis in my mind.”* |
| Facilitating personal reflection | Mentions Future Me helping facilitate a self-questioning about their values, motivation and goals | 80% | *“I do quite like the way it was sort of prompting me to sort of think about my future in a way. So, it asked me questions like, okay, how do you feel about this? And about this opportunity? And what are your goals, for example. So, it was quite interesting how it sort of prompted, I guess, thoughts on my part.”* |
| New knowledge, perspectives and ideas | Mentions Future Me providing them with new  knowledge or ideas to help them make  decisions or work towards a goal | 70% | *“I need to actually look for  programs that are APA-certified, you know, things that I didn't really realise before and that’s helpful  to look into. Now, I have like  specific things to move forward in”* |
| Reaffirming and consolidating | Mentions finding the conversation with Future Me reaffirming, reassuring or  Future Me helping them to reconsolidate  and remind them of existing knowledge and advice. | 65% | *“I was reminded of things I feel like it's more just revision, almost, consolidation, yeah, consolidation because it's answering a specific question, it puts everything  related to that question together so that's more, maybe the helpful part”* |
| Comparison to human conversation | Mentions Future Me helping them to question  their own values, intentions or desires  about the future. | 65% | *“Everything was kind of connected to what I was saying, and there was  some, some kind of objectively good advice that it was the kind of thing that you imagine someone who actually was human would  give you”* |
| Text message format | Mentions liking the text message format  of Future Me | 50% | *“Phone texting is already a mechanism people are used to so it's not something additional like for me it’s like a very natural thing to do, like text and receive answers it was just very easy to use”* |

Table 5: Negative themes found in the user feedback about FutureMe

| **Theme** | **Description** | **Frequency of presence** | **Example (codes only)** |
| --- | --- | --- | --- |
| **Negative Perceptions of Future Me** |  |  |  |
| User dependence | Mentions that they dislike or find it difficult  that Future Me is primarily led by their  messages rather than Future Me | 70% | *“I think the chatbot was kind of lazy. I think most of the burden was on me to think about my future, like to kind of prime it, or to guide the conversation.”* |
| Formulaic | Mentions that Future Me’s responses  are formulaic, generic and predictable | 60% | *“It also felt a bit automatic because a lot of the advice that I got was very similar to each other. It repeated a bunch of things, not like actual sentences, but like advice, things that I should do.”* |
| Lacking something human | Mentions that they found the interaction with Future Me to be lacking something inherently human, such as lacking intention or subjective experience | 50% | *“While it may be helpful, I don't know how much I could fully buy into it, just with the knowledge that it wasn't  a living and breathing person at the other end of the phone so there might be less trust because I think when it  comes to the advice.”* |
| Conversation flow | References that conversation flow with Future Me was somehow problematic for them | 45% | *“There was a bit of like a lag in it's like immediate response I felt like I was using it wrong like that something I had said was making it like malfunction so I was kind of like second guessing myself Or I thought that maybe like my phone signal had died,  or something, so it wouldn't be able to respond.”* |

Table 6: Themes revealed from the thematic analysis of the baseline interview transcripts (N=14)

| **Theme** | **Description** | **Frequency** | **Example** |
| --- | --- | --- | --- |
| Trust and  Emotional  Openness | This theme captures the complex relationship between trust, emotional connection, and the use of chatbots. It includes concerns about the lack of human warmth and the potential impact of university affiliations on trust, as well as the positive influence of anonymity and peer experiences on openness and trust. | 50% | *“I’m not sure how a digital tool could replicate the compassionate side [of humans]…if you’re emotional and you’re crying, people can understand that and comfort you, you know a human would give you a tissue or whatever, whether they [chatbots] could match that would be a concern of mine”* |
| Role and Effectiveness | This theme explores how students perceive the role of chatbots in their stress management strategies, especially compared to human support. It includes the perceived advantages of chatbots (e.g., objectivity, immediacy) and the limitations (e.g., emotional understanding, compassion). | 43% | *“A digital tool can be useful when you are normally stressed out. But when you are very stressed, you might want to look for a real person to help…my mom or my friends know what kind of person I am, what I need, what type of comfort I need. When it comes to a digital tool, they are quite general…so that’s where the disconnect might come from.”* |
| Personalisation and Contextual Awareness | This theme focuses on the students' desire for personalised and contextually aware chatbot interactions. It highlights the need for chatbots to be knowledgeable about the students' specific academic pressures and stressors and to tailor their responses accordingly. | 43% | *“If [chatbot] had background knowledge on her course it would know that I have deadlines or a dissertation coming up and it would know that I might be more stressed around those dates. So maybe the digital tool will be like, ‘hey are you okay?’, which is basically the same as my friends asking me ‘how is your dissertation going?’”* |

Table 7 Themes identified from the thematic analysis of participants’ inputs during conversations with FutureMe (N=12).

| **Theme** | **Description** | **Frequency** | **Example** |
| --- | --- | --- | --- |
| Academic stress: quick tips for stress relief, time management advice | They expressed stress related to their academic responsibilities, including the pressure of dissertation deadlines, their research workload and their performance in exams and presentations. They sought quick tips for stress relief, as well as practical advice on how to manage their time across competing priorities and break down projects into smaller, more manageable tasks. | 75% | *“I’m nervous because I think I’m not well prepared for the presentation but it is so hard that I feel unbearable sitting here thinking about it. That’s why I’m not sure about the idea of getting some rest – I’m afraid that I will be wasting my time.”* |
| Negative emotions and low motivation: practical tips to boost motivation, emotional validation, help understanding emotions | They described negative emotions and struggles with their mental well-being, including feelings of anxiety, sadness and a lack of motivation. They sought practical tips on how to improve their motivation, as well as validation for their negative emotions and information to help them understand the potential reasons behind those emotions. | 58% | *“Can you help me figure out why I am sad?”* |
| Future uncertainty: advice on handling uncertainties, self-reflection | The uncertainty about the future, particularly concerning job prospects and long-term plans, was a major source of stress. They sought guidance on managing these uncertainties and reflected on their goals and values to make informed decisions and consider potential solutions to their future concerns. | 58% | *“I am stressed about leaving the country this summer and getting a job after graduation.”* |
| Social relationships: coping advice, friendship advice | Social relationships were another area of stress. International students who are newer to the UK expressed concerns around making new friends. They sought advice on how to maintain previous connections and cope with their loneliness. | 25% | *“I don’t know how to make friends at the pool…I usually feel too shy to talk to other people until they speak to me first.”* |

Table 8: Themes revealed from the thematic analysis of the follow-up interview transcripts (N=12)

| **Theme** | **Description** | **Frequency** | **Example** |
| --- | --- | --- | --- |
| Accessible and convenient | Participants appreciated that FutureMe provided easily accessible, persistent support, especially during off-hours when friends, family, or professional help were unavailable. This accessibility allowed them to manage stress at their own pace without feeling like they were burdening others or that their stress wasn’t significant enough to seek human support. Many participants noted that having a tool available 24/7 gave them autonomy in managing their well-being, particularly when traditional mental health services were limited to standard office hours. | 58% | *"If you're stressed even in the night then you know that this [FutureMe] would respond to you as opposed to a friend that's fallen asleep…it gives you the option to manage your stress at all hours as opposed to just at 5:00 PM. I know a lot of mental health professional systems are nine to five, or ‘eight to four we’ll have a live chat system’, as opposed to something that is 24 hours a day."* |
| Personalized and relatable | Participants valued the personalized interactions provided by FutureMe, which helped them feel more engaged and comfortable. They appreciated that the chatbot’s conversational, friendly tone made interactions feel less formal and more like a conversation with a peer. Many participants noted that the responses felt tailored and relevant, which encouraged further use of the chatbot over time. However, there were mixed reactions regarding the depth of personalization, with some participants noting that over time, responses started to feel somewhat repetitive. | 42% | *“It almost felt like it [FutureMe] had a mind of its own which felt nice, it didn’t feel very scripted. It actually felt like it was able to understand what I was saying and respond with relevant questions.”* |
| Need better balance between practical advice and emotional support | Participants appreciated that FutureMe provided easily accessible, persistent support, especially during off-hours when friends, family, or professional help were unavailable. This accessibility allowed them to manage stress at their own pace without feeling like they were burdening others or that their stress wasn’t significant enough to seek human support. Many participants noted that having a tool available 24/7 gave them autonomy in managing their well-being, particularly when traditional mental health services were limited to standard office hours. | 58% | *"If you’re stressed even in the night then you know that this [FutureMe] would respond to you as opposed to a friend that’s fallen asleep. . . it gives you the option to manage your stress at all hours as opposed to just at 5:00 PM. I know a lot of mental health professional systems are nine to five, or ‘eight to four we’ll have a live chat system’, as opposed to something that is 24 hours a day."* |
| Want responses that are more tailored to the current context and stress level | Participants expressed a strong desire for FutureMe’s responses to be more context-sensitive and responsive to their immediate stress levels. They suggested that the chatbot ask fewer questions during periods of intense stress and offer more support that aligned with their emotional needs at the time. Many participants felt that a voice-based interaction could enhance the sense of personalization, especially when they were in a frantic or emotional state, as typing felt time-consuming and less expressive. Voice input, they believed, would allow them to convey their emotions more naturally and feel a greater connection to the chatbot | 58% | *“Having a voice would feel more comforting and would make it easier to talk about how you feel, especially if you just wanna get stuff off your chest. Typing can take ages and you can miss out a lot of stuff in the interest of time, but if you’re talking that would be very helpful. . . If you’re in a very frantic mood, the voice would definitely be better because it’s hard to convey how you feel in that moment over text.”* |
| Envision integration with human support systems | While participants saw FutureMe as a helpful tool for initial stress relief, they viewed it as complementary to, rather than a replacement for, human support. They felt that FutureMe’s role should involve offering practical advice and guiding them toward human help when needed, especially during crises. Participants expressed the need for FutureMe to integrate more seamlessly with university mental health services or external resources, offering clear pathways to professional help when the chatbot’s capabilities were insufficient. | 67% | *“If something was more serious and someone wanted to seek professional help, then perhaps a link or an avenue or number for help, whether this is through your university or not, would be good because it would allow you to think, OK, maybe I do need help. . . it opens the door of professional help if someone does need it”* |
